# Antioxidant and Cytoprotective Effects of the Di-*O*-Caffeoylquinic Acid Family: The Mechanism, Structure–Activity Relationship, and Conformational Effect

**DOI:** 10.3390/molecules23010222

**Published:** 2018-01-20

**Authors:** Xican Li, Ke Li, Hong Xie, Yulu Xie, Yueying Li, Xiaojun Zhao, Xiaohua Jiang, Dongfeng Chen

**Affiliations:** 1 School of Chinese Herbal Medicine, Guangzhou University of Chinese Medicine, Waihuan East Road No. 232, Guangzhou Higher Education Mega Center, Guangzhou 510006, China; xiehongxh1@163.com (H.X.); xieyulu1900@163.com (Y.X.); lln@gzucm.edu.cn (Y.L.); zxj@gzucm.edu.cn (X.Z.); 2 Innovative Research & Development Laboratory of TCM, Guangzhou University of Chinese Medicine, Waihuan East Road No. 232, Guangzhou Higher Education Mega Center, Guangzhou 510006, China; 3 School of Basic Medical Science, Guangzhou University of Chinese Medicine, Waihuan East Road No. 232, Guangzhou Higher Education Mega Center, Guangzhou 510006, China; ys1090992678@163.com; 4 The Research Center of Basic Integrative Medicine, Guangzhou University of Chinese Medicine, Waihuan East Road No. 232, Guangzhou Higher Education Mega Center, Guangzhou 510006, China; 5 School of Biomedical Sciences, Faculty of Medicine, The Chinese University of Hong Kong, Sha Tin, Hong Kong 999077, China; xjiang@cuhk.edu.hk

**Keywords:** conformational effect, caffeoylquinic acids, antioxidant, cytoprotective effect

## Abstract

In this study, a series of di-*O*-caffeoylquinic acids (**di-COQs**) were systematically investigated for their antioxidant and cytoprotective effects towards •OH-damaged bone marrow-derived mesenchymal stem cells (bmMSCs). Five **di-COQs** were measured using a set of antioxidant assays. The results show that adjacent 4,5-Di-*O*-caffeoylquinic acid (**4**,**5-COQ**) and 3,4-di-*O*-caffeoylquinic acid (**3**,**4-COQ**) always gave lower IC_50_ values than did non-adjacent **di-COQs**. In the Fe^2+^-chelating assay, **4**,**5-COQ** and **3**,**4-COQ** presented greater UV-Vis spectra and darker colors than did non-adjacent **di-COQs**. In the UPLC-ESI-MS/MS analysis, no corresponding radical adduct formation (RAF) peak was found in the reaction products of **di-COQs** with PTIO•. In the MTT assay, all **di-COQs** (especially **1,5-COQ**, **1,3-COQ**, and **4**,**5-COQ**) dose-dependently increased the cellular viabilities of •OH-damaged bmMSCs. Based on this evidence, we conclude that the five antioxidant **di-COQs** can protect bmMSCs from •OH-induced damage. Their antioxidant mechanisms may include electron-transfer (ET), H^+^-transfer, and Fe^2+^-chelating, except for RAF. Two adjacent **di-COQs** (**4**,**5-COQ** and **3**,**4-COQ**) always possessed a higher antioxidant ability than the non-adjacent **di-COQs** (**1**,**3-COQ**, **1**,**5-COQ**, and **3**,**5-COQ**) in chemical models. However, non-adjacent **1**,**3-COQ** and **1**,**5-COQ** exhibited a higher cytoprotective effect than did adjacent **di-COQs.** These differences can be attributed to the relative positions of two caffeoyl moieties and, ultimately, to the conformational effect from the cyclohexane skeleton.

## 1. Introduction

The activity of synthetic and natural antioxidants is derived from the molecular phenolic moiety, but it can be affected by structural factors such as hydrogen-bonding [1], the amount of phenolic –OHs [2,3], *O*-methylation [4], glycosidation [5], anisylation [6], and heterocycles [6]. Even the 6″-OH group of the sugar residue in flavonoid glycosides could alter the antioxidant levels [7].

However, these structural factors are limited to molecular “structure” and are not involved in the molecular “conformation.” From the perspective of organic chemistry [8], molecular conformation derives from the ***σ***-bond free rotation, which can alter the spatial relative position of the moieties (or atoms). A representative example is the cyclohexane molecule, which can give two distinctive conformations: chair conformation and boat conformation. The chair conformation has been proven to be preferential; in it, the axial bond (***a*** bond) and equatorial bond (***e*** bond) are alternately arrayed. The difference between the ***a*** bond and ***e*** bond results in the chemical characteristics of the moieties (or atoms), which has been called the conformational effect. Recently, the conformational effect has been found to change some of the chemical properties, such as red-shifted emission, of luciferin [9]. It is hypothesized that, if an antioxidant moiety is attached to the cyclohexane skeleton and occupies different bond types (***a***/***e ***bonds), its antioxidant potential may be distinctive. However, such conformational effects towards antioxidant ability have not, to our knowledge, yet been reported.

**Figure 1:**
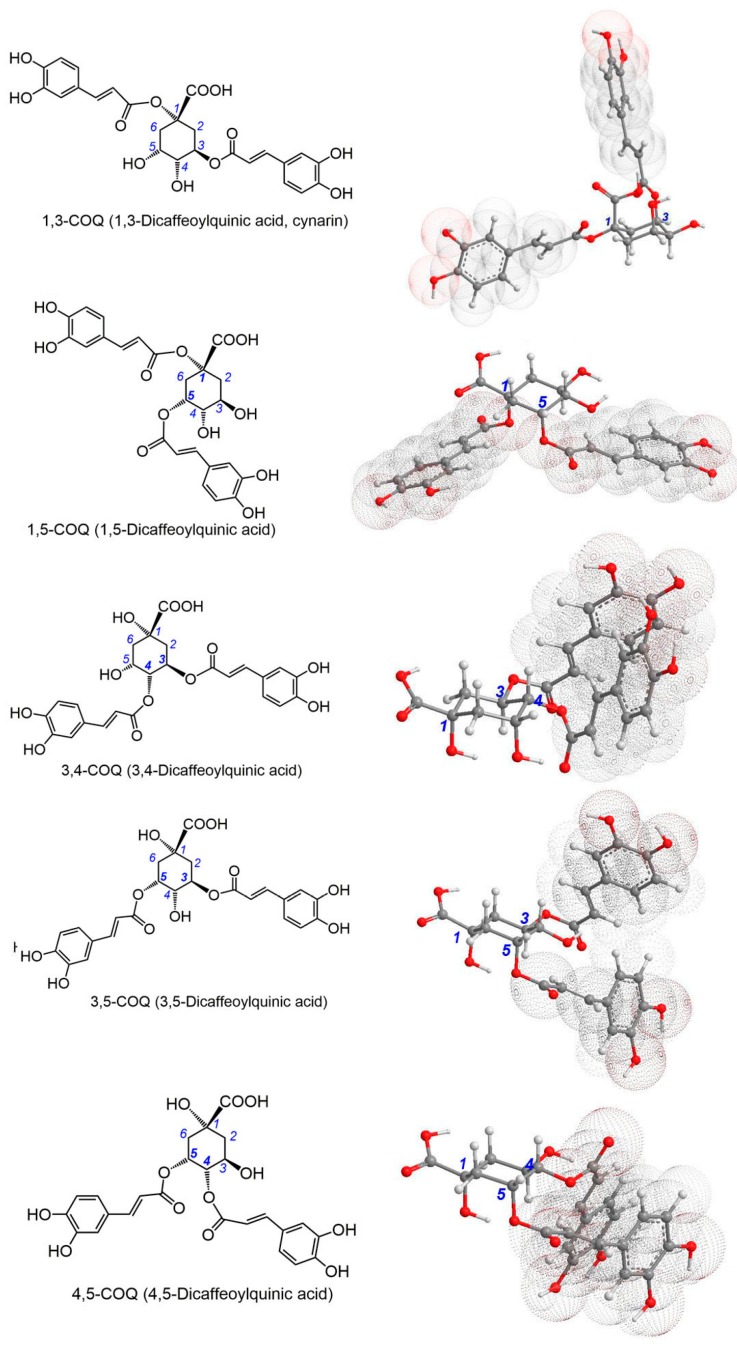
Structures (**left**) and preferential conformation-based ball-stick models (**right**) of five di-*O*-caffeoylquinic acids (**di-COQs**). The ball-stick models were created in Chem3D Pro 14.0. The screenshots from models are from the same perspective; i.e., C-1 was deposited on the right end, and –COOH is upward. The three-dimensional perspective animations are shown in Video S1–5. However, the relative degree of crowd for caffeoyl moieties remains unchanged.

In this study, five di-*O*-caffeoylquinic acids (**di-COQ**), which are distributed in different plants [10,11,12,13,14], were selected as references for the conformational effect investigation. As shown in Figure 1, the five **di-COQs** comprise 1,3-di-*O*-caffeoylquinic acid (**1**,**3-COQ**), 1,5-di-*O*-caffeoylquinic acid (**1**,**5-COQ**), 3,4-di-*O*-caffeoylquinic acid (**3**,**4-COQ**), 3,5-di-*O*-caffeoylquinic acid (**3**,**5-COQ**), and 4,5-di-*O*-caffeoylquinic acid (**4**,**5-COQ**). In the five molecules, the caffeoyl moiety, with antioxidant potential, is attached to hexacyclic quinic acid; thus, these five acids may be the ideal reference for such an investigation.

It is worth noting that some of these compounds have already been explored for their antioxidant ability using a DPPH•-scavenging assay, ABTS•^+^-scavenging assay, anti-low-density lipoprotein (LDL) oxidation assay [11,15], and cellular assay [13]. However, each of these studies was based on the plant origin: Hung focused on antioxidants from *Dipsacus asper*, Zhang only explored antioxidants in *Lonicera japonica*, and Wan was only engaged in the phytochemical work of *Chrysanthemum coronarium* [14]. Thus, for **di-COQs**, these works are non-systematical. For instance, the phytochemical work of Wan lacks **1**,**3-COQ** [14] and is irrelevant to the antioxidant study, and Hung’s work lacks two important members: **1**,**3-COQ** and **1**,**5-COQ**. However, some mono-*O*-caffeoylquinic acids (e.g., chlorogenic acid) and flavonoids were involved in these studies [10,11,14]. Hence, these works are non-comparative and cannot be used to analyze the structure–activity relationship of the **di-COQs** antioxidant family.

The present study, however, used five **di-COQs** for comparative study, based on chemical and cellular models. The cellular model is based on oxidatively stressed bone marrow-derived mesenchymal stem cells (bmMSCs). bmMSCs are considered a highly promising cell type candidate for cell-based tissue transplantation engineering and regeneration, but they are limited by their lower cellular viability derived from oxidative stress [16]. Our study will also provide new information about the **di-COQs** family regarding bmMSCs transplantation engineering.

## 2. Results and Discussion

Iron overload can induce oxidative stress to severely damage cells, which can cause a series of diseases (including neurodegeneration), resulting from the ability of iron (particularly Fe^2+^) to promote the generation of ROS [17]. A typical example is the Fenton reaction, which can produce •OH radicals. Thus, iron chelation has now been developed as a therapy for these diseases [18,19], and the iron chelation level of a natural antioxidant is regularly evaluated using colorimetric methods and UV-Vis spectra analysis [20,21]. However, UV-Vis spectra analysis is considered direct evidence of an iron chelating reaction [22,23].

**Figure 2:**
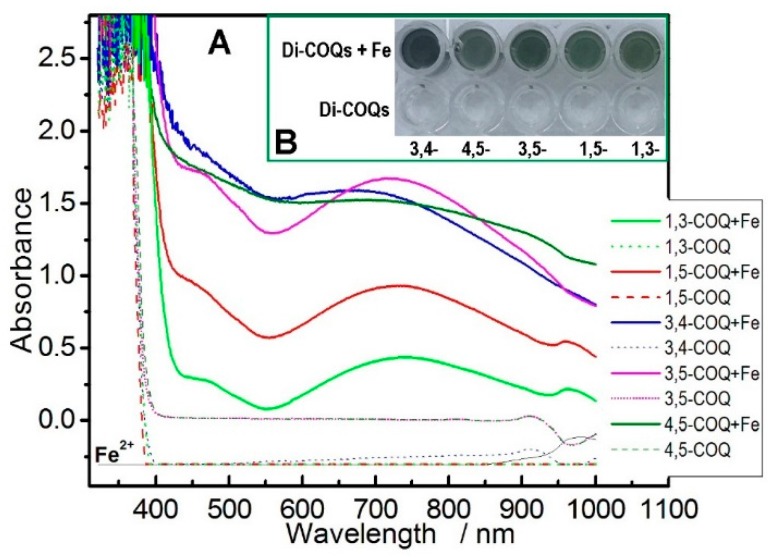
(**A**) UV-Vis spectra of the five **di-COQs** and their chelating products with excess Fe^2+^. (**B**) The colors of complexes, resulting from the product mixtures, were taken by a camera.

**Figure 3:**
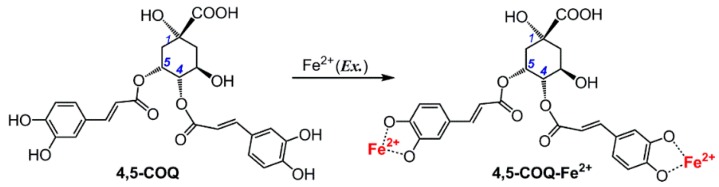
The proposed chelating reaction of **4**,**5-COQ** (4,5-di-*O*-caffeoylquinic acid) with excessive Fe^2+^ (the reaction formula is proposed based on previous studies [11,22,23,24]).

In the present study, we used UV-Vis spectra to analyze the Fe^2+^-chelating ability of the five **di-COQs**. As shown in Figure 2, after incubation with Fe^2+^, each of the five **di-COQs** gave rise to an absorption maximum around 750 nm and a green product mixture, suggesting that an Fe^2+^-chelating reaction between Fe^2+^ and each of the **di-COQs** occurs. Therefore, each of the **di-COQs** may undergo an Fe^2+^-chelating approach to reduce the oxidative stress from ROS (especially •OH). A typical Fe^2+^-chelating reaction could be proposed, as shown in Figure 3. Since Fe^2+^-chelating can indirectly release oxidative stress, it is sometimes called the indirect antioxidant mechanism.

Correspondingly, radical-scavenging is termed a direct antioxidant mechanism. In this study, five **di-COQs** were observed to dose-dependently scavenge various radicals in chemical models, including PTIO•, DPPH•, and ABTS^+^• radicals (Figure S1). PTIO• is an oxygen-centered radical, whereas both DPPH• and ABTS^+^• are nitrogen-centered radicals. The ability of the five **di-COQs** to scavenge the three radicals implies that they can scavenge not only ROS but also reactive nitrogen species (RNS, e.g., ONOO^−^ and NO) in cells and may undergo a direct antioxidant approach to reduce the oxidative stress.

Furthermore, these free radical-scavenging reactions are mediated by different antioxidant pathways. PTIO• scavenging at pH 4.5 is an electron transfer (ET) pathway [25]. The effectiveness of the five **di-COQs** with PTIO• scavenging at pH 4.5 shows that they can undergo ET to exert their antioxidant action, which is further supported by evidence from the FRAP assay, a mere ET process [26].

However, PTIO• scavenging at pH 7.4 was proven to be an H^+^-transfer pathway [27]. Since the five **di-COQs** can efficiently scavenge PTIO• at pH 7.4, this implies that a H^+^-transfer may play a role in their antioxidant action. It is worth noting that the so-called ET or H^+^-transfer is a unidirectional process, where the antioxidant donates an electron or H^+^ to the radical rather than the antioxidant accepting an electron or H^+^ from the radical [21,27,28].

Unlike the FRAP assay (or PTIO• scavenging at pH 4.5), DPPH•-scavenging and ABTS^+^•-scavenging assays are mediated via complicated pathways. DPPH•-scavenging includes multiple hydrogen atom transfer (HAT)-based pathways [27], and ABTS^+^• is scavenged through multiple ET-based pathways [26]. The DPPH•-scavenging and ABTS^+^•-scavenging by the five **di-COQs** indicated that their antioxidant actions may also be fulfilled via multiple antioxidant pathways, including H^+^-transfer, ET, and HAT.

It should be noted that the radical adduct formation (RAF) may also occur in the radical-scavenging action of phenolic antioxidants [28,29]. However, no RAF product peak was observed in the UPLC-ESI-MS/MS spectra of the **di-COQs** reaction products with PTIO•. By comparison, chlorogenic acid (a mono-*O*-caffeoylquinic acid) generated a peak at *m*/*z* 708, which is the value of the chlorogenic acid–chlorogenic acid dimer (Figure S2). This suggests that the five **di-COQs** cannot mediate RAF to exert the antioxidant action. The inactivity of **di-COQs** in the RAF pathway is presumed to be from steric hindrance, although this presumption needs further identification. Therefore, the evidence from the chemical models indicated that as natural antioxidants, **di-COQs** may undergo multiple antioxidant pathways (including H^+^-transfer, ET, or HAT, but not RAF) to exert their antioxidant action.

From the perspective of quantitative analysis, the IC_50_ values of the five **di-COQs** were different from each other (Table 1), which indicates that there are differences in the relative antioxidant levels. In general, adjacent **di-COQs** (**4**,**5-COQ** and **3**,**4-COQ**) always possess higher levels than do non-adjacent **di-COQs** (**1**,**3-COQ**, **1**,**5-COQ**, and **3**,**5-COQ**). Interestingly, the relative levels are similar to the anti-inflammatory activities [30].

As shown in Figure 1 (right), **4**,**5-COQ** and **3**,**4-COQ** contain two adjacent caffeoyl moieties and belong to adjacent **di-COQs**. Caffeoyl moieties are attached to the chair conformation-preferred hexacyclic skeleton, where the ***a*** and ***e*** bonds are alternately arrayed [9]. In **4**,**5-COQ**, two caffeoyl moieties present a *trans*-configuration; in **3**,**4-COQ**, however, two caffeoyl moieties display a *cis*-configuration. Despite having two adjacent caffeoyl moieties in a *trans*-configuration and an ***e*** bond, they are still very crowded. The degree of crowd increases the molecular energy, thereby elevating the redox potential. Thus, in the redox-based antioxidant assays, **4**,**5-COQ** and **3**,**4-COQ**, which contain two adjacent caffeoyl moieties, are always more effective than are the three non-adjacent **di-COQs** (**1,3-COQ**, **1,5-COQ**, and **3,5-COQ**). In each of the three non-adjacent **di-COQs**, two caffeoyl moieties are distant from each other, regardless of the ***a***/***e*** bonds and *trans*-/*cis*- configurations. The distance effectively releases the crowd to decrease the molecular energy and redox reactivity. Correspondingly, the antioxidant potential has been lowered.

The difference between adjacent and non-adjacent **di-COQs** can also be observed in the Fe^2+^-chelating assay. As shown in Figure 2A, compared by three non-adjacent **di-COQs**, both **4**,**5-COQ** and **3**,**4-COQ** gave stronger peaks in the UV spectra when treated by excessive Fe^2+^. In the aspect of complex color, **4**,**5-COQ** and **3**,**4-COQ** also yielded a darker color than did the three non-adjacent **di-COQs** (Figure 2B). These results imply that adjacent **4**,**5-COQ** and **3**,**4-COQ** also displayed a higher Fe^2+^-chelating ability than did the three non-adjacent **di-COQs**.

In each member of the **di-COQs** family, the ligand for the Fe^2+^-chelating reaction is the caffeoyl moiety [11]. As shown in Figure 1 (right), two caffeoyl moieties in **3**,**4-COQ** and **4**,**5-COQ** molecules stretch out of the skeleton on the same side, in which they can surround excessive Fe^2+^ to participate in the chelating reaction. In contrast, two caffeoyl moieties in non-adjacent **di-COQs** (especially **1**,**3-COQ** and **1**,**5-COQ**) extended from two directions and can hardly surround excessive Fe^2+^ for joint chelation. As a result, **3**,**4-COQ** and **4**,**5-COQ** are more effective Fe^2+^-chelators than are the three non-adjacent **di-COQs**. It should be noted that the dihydroxyl groups in the quinic acid ring cannot chelate metals to form a stable ringed complex, such as the 4,5-dihydroxyl groups in **1**,**3-COQ** and the 3,4-dihydroxyl groups in **1**,**5-COQ** [21,23,31]. In a word, two adjacent **di-COQs** (**4**,**5-COQ** and **3**,**4-COQ**) always possess a higher antioxidant ability than the three non-adjacent **di-COQs** (**1**,**3-COQ**, **1**,**5-COQ**, and **3**,**5-COQ**) in chemical models.

In the cellular model, their relative cytoprotective levels exhibit only small changes. As shown in Table 2, all five **di-COQs** could concentration-dependently enhance the viability percentages of •OH-treated bmMSCs in the MTT assay. Furthermore, **1**,**3-COQ**, **1**,**5-COQ**, and **4**,**5-COQ** gave higher viability percentages than did the other compounds. Generally speaking, two non-adjacent **di-COQs** (**1**,**3-COQ** and **1**,**5-COQ**) exhibit a higher cytoprotective effect than adjacent **di-COQs**. It is hypothesized that **1**,**3-COQ** and **1**,**5-COQ** exhibited a better cytoprotective effect due to their molecular shapes. In **1**,**3-COQ** and **1**,**5-COQ**, two caffeoyl moieties extend from the molecule in different directions, resulting in a long and narrow structure, which may help them freely cross the cytomembrane to the nucleus. However, the above hypothesis requires further study. In short, these differences among the five **di-COQs** in terms of antioxidant or cytoprotective effects may result from the conformation of the coral cyclohexane skeleton.

## 3. Materials and Methods

### 3.1. Chemicals and Animals

1,3-Di-*O*-caffeoylquinic acid (CAS 30964-13-7, 97%), 1,5-di-*O*-caffeoylquinic acid (CAS 19870-46-3, 97%), 3,4-di-*O*-caffeoylquinic acid (CAS 14534-61-3, 97%), 3,5-di-*O*-caffeoylquinic acid (CAS 2450-53-5, 97%), and 4,5-di-*O*-caffeoylquinic acid (CAS 57378-72-0, 97%) were obtained from Chengdu Biopurify Phytochemicals Ltd. (Chengdu, China). The 1,1-diphenyl-2-picryl-hydrazl radical (DPPH•), (±)-6-hydroxyl-2,5,7,8-tetramethlychromane-2-carboxylic acid (Trolox), 2,4,6-tripyridyltriazine (TPTZ), and 3-(4,5-dimethylthiazol-2-yl)-2,5-diphenyltetrazolium bromide (MTT) were purchased from Sigma-Aldrich Shanghai Trading Co. (Shanghai, China). (NH_4_)_2_ABTS [2,2′-azino-bis(3-ethylbenzo-thiazoline-6-sulfonic acid diammonium salt)] was obtained from Amresco Chemical Co. (Solon, OH, USA). The 2-phenyl-4,4,5,5-tetramethylimidazoline-1-oxyl-3-oxide radical (PTIO•) was from TCI Chemical Co. (Shanghai, China). Dulbecco’s modified Eagle’s medium (DMEM), fetal bovine serum (FBS), and trypsin were purchased from Gibco (Grand Island, NY, USA). CD44 and Proteinase K were purchased from Wuhan Boster Co., Ltd. (Wuhan, China). All other reagents were of analytical grade.

Sprague–Dawley (SD) rats of 4 weeks of age were obtained from the Animal Center of Guangzhou University of Chinese Medicine (Guangzhou, China). The protocol of this experiment was performed under the supervision of the Institutional Animal Ethics Committee in Guangzhou University of Chinese Medicine (Approval number 20170306A).

### 3.2. UV-Vis Spectra Analysis of Fe^2+^-Chelating with **di-COQs**

This method was based on the previous study [20]. Briefly, 100 μL of a methanolic solution of **di-COQs** (3 mg/mL) was added to 400 μL of an aqueous solution of FeCl_2_·4H_2_O (10 mg/mL). The solution was then mixed vigorously. Subsequently, the resulting mixture was incubated at room temperature for 30 min, and the spectrum was obtained using a UV-Vis spectrophotometer (Jinhua 754 PC, Shanghai, China) from 200–1000 nm. Then, 200 μL of the supernatant was transferred to a 96-well plate and photographed using a camera.

### 3.3. PTIO•-Scavenging Assay

The PTIO•-scavenging assay was conducted based on our method [32]. In brief, 80 μL of an aqueous PTIO• solution (0.1 mM) was mixed with 20 μL of phosphate buffer (pH 4.5, 7.4) containing sample (5 mg/mL) at the indicated concentrations. The mixture was maintained at 37 °C for 2 h, and the absorbance was measured at 560 nm on a microplate reader (Multiskan FC, Thermo Scientific, Shanghai, China). The PTIO• inhibition percentage was calculated as follows:

(1) $$Scavenging\%=\frac{A_{0}-A}{A_{0}}$$

where *A*_0_ indicates the absorbance of the blank and *A* indicates the absorbance of the sample.

### 3.4. FRAP Assay

The FRAP assay was established by Benzie and Strain [33]. In the present study, the FRAP reagent was prepared freshly by mixing 10 mM TPTZ, 20 mM FeCl_3_, and 0.25 M acetate buffer (pH 3.6) at 1:1:10. The sample solution (x = 1–9 μL, 0.1 mg/mL) was added to (20 − x) μL of 95% ethanol followed by 80 μL of FRAP reagent. After incubation at ambient temperatures for 30 min, the absorbance was measured at 595 nm using distilled water as the blank. The relative reducing power of the sample was calculated using the formula:

(2) $$Relative\;reducing\;effect\%=\frac{A-A_{\min}}{A_{\max}-A_{\min}}$$

where *A*_max_ is the maximum absorbance, and *A*_min_ is the minimum absorbance in the test. *A* is the absorbance of sample.

### 3.5. DPPH•-Scavenging Assay

DPPH• radical-scavenging activity was determined as previously described [34]. Briefly, 80 μL of DPPH• solution (0.1 mol/L) was mixed with the indicated concentrations of sample (0.05 mg/mL, 2–10 μL) dissolved in methanol. The mixture was maintained at room temperature for 30 min, and the absorbance was measured at 519 nm on a microplate reader. The percentage of DPPH• scavenging activity was calculated based on the formula presented in Section 3.3.

### 3.6. ABTS•^+^-Scavenging Assay

The ABTS•^+^-scavenging activity was evaluated according to the method [24]. The ABTS•^+^ was produced by mixing 0.2 mL of (NH_4_)_2_ABTS (7.4 mmol/L) with 0.35 mL of potassium persulfate (2.6 mmol/L). The mixture was kept in the dark at room temperature for 12 h to allow completion of radical generation and then diluted with distilled water (about 1:20), so that its absorbance at 734 nm was measured on a microplate reader. To determine the scavenging activity, the test sample (x = 1–9 μL, 0.1 mg/mL) was added to (20 − x) μL of distilled water followed by 80 μL of ABTS•^+^ reagent, and the absorbance at 734 nm was measured 3 min after the initial mixing, using distilled water as the blank. The percentage inhibition of the samples was calculated based on the formula listed in Section 3.3. 

### 3.7. UPLC-ESI-Q-TOF-MS/MS Analysis of Reaction Products of **di-COQs** and Chlorogenic Acid with PTIO•

This method was based on the previous study [27]. The methanol solution of **di-COQs** was mixed with a solution of PTIO• radical in methanol at a molar ratio of 1:2, and the resulting mixture was incubated for 24 h at room temperature. The product mixture was then filtered through a 0.22 μm filter and analyzed using a UPLC-ESI-Q-TOF-MS/MS system equipped with a C_18_ column (2.0 mm i.d. × 100 mm, 2.2 μm, Shimadzu Co., Kyoto, Japan). The mobile phase was used for the elution of the system and consisted of a mixture of methanol (Phase A) and water (Phase B). The column was eluted at a flow rate of 0.3 mL/min with the following gradient elution program: 0–10 min, 60%–100% A; 10–15 min, 100% A. The sample injection volume was set at 1 μL for the separation of the different components. Q-TOF-MS/MS analysis was performed on a Triple TOF 5600*^plus^* Mass spectrometer (AB SCIEX, Framingham, MA, USA) equipped with an ESI source, which was run in the negative ionization mode. The scan range was set at 100–2000 Da. The system was run with the following parameter: ion spray voltage: −4500 V; ion source heater: 550 °C; curtain gas (CUR, N_2_): 30 psi; nebulizing gas (GS1, Air): 50 psi; Tis gas (GS2, Air): 50 psi. The declustering potential (DP) was set at −100 V, whereas the collision energy (CE) was set at −40 V with a collision energy spread (CES) of 20 V. The RAF products were quantified by extracting corresponding formula (e.g., [C_43_H_36_N_5_O_18_−H]^−^ for **di-COQs**-DPPH•) from the Total Ion Chromatogram, integrating the corresponding peak. We used chlorogenic acid as a positive control to repeat the above experiments, instead of **di-COQs**.

### 3.8. Cytoprotective Effect towards •OH-Damaged bmMSCs (MTT Assay)

The bmMSCs were cultured according to our previous report [35] with slight modifications. In brief, bone marrow was obtained from the femur and tibia of rat. The marrow samples were diluted with DMEM (low glucose) containing 10% FBS. The bmMSCs were prepared by gradient centrifugation at 900 *g* for 30 min on 1.073 g/mL Percoll. The prepared cells were detached by treatment with 0.25% trypsin and passaged into cultural flasks at 1 × 10^4^/cm^2^. The bmMSCs at Passage 3 were evaluated for cultured cell homogeneity.

**Figure 4:**
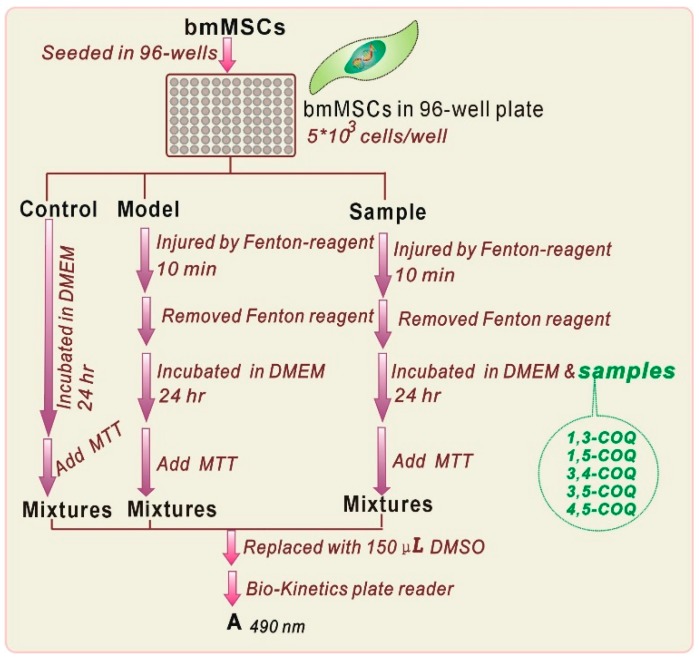
Experimental procedures for the MTT assay. (PE-1420 Bio-Kinetics reader: Bio-Kinetics Corporation, Sioux Center, IA, USA. Each test was repeated in five independent wells. MTT was used at 5 mg/mL (in PBS), and the addition volume was 20 μL. The addition of Fenton reagent was conducted by injection of FeCl_2_ (100 μM) followed by H_2_O_2_ (50 μM).

The MTT assay was used to evaluate cytoprotective effect of **di-COQs** towards bmMSCs [34,36,37]. The experimental protocol is briefly illustrated in Figure 4.

### 3.9. Statistical Analysis

Each experiment was performed in triplicate and the data were recorded as mean ± SD (standard deviation). The dose response curves were plotted using Origin 6.0 professional software (OriginLab, Northampton, MA, USA). The IC_50_ value was defined as a final concentration of 50% radical inhibition (or relative reducing power) [38]. Statistical comparisons were made by one-way ANOVA to detect significant difference using SPSS 13.0 (SPSS Inc., Chicago, IL, USA) for Windows. *p* < 0.05 was considered to be statistically significant.

## 4. Conclusions

Five antioxidant **di-COQs** can protect bmMSCs from •OH-induced damage. Their antioxidant mechanisms may include ET, H^+^-transfer, and Fe^2+^-chelating, except for RAF. However, the antioxidant (or cytoprotective) levels are different among them. These differences can be attributed to the positions of the two caffeoyl moieties and, ultimately, to the conformational effect from the cyclohexane skeleton.

## Figures and Tables

**Table 1:** The IC_50_ values of five **di-COQs** in various antioxidant assays.

Compounds	PTIO•-Scavenging (pH 4.5, mg/mL, mM)	PTIO• Scavenging (pH 7.4, mg/mL, mM)	FRAP (μg/mL, μM)	DPPH•-Scavenging (μg/mL, μM)	ABTS^+^•-Scavenging (μg/mL, μM)
**1,3-COQ**	47.2 ± 1.6	57.7 ± 1.0	3.4 ± 0.2	2.9 ± 0.1	3.6 ± 0.0
	(91.4 ± 3.6 ^e^)	(111.8 ± 2.0 ^c^)	(6.5 ± 0.4 ^c^)	(5.7 ± 0.3 ^b^)	(6.9 ± 0.1 ^c^)
**1,5-COQ**	35.5 ± 2.8	63.0 ± 7.6	3.3 ± 0.1	4.7 ± 0.6	3.5 ± 0.0
	(68.7 ± 5.4 ^d^)	(121.9 ± 14.8 ^c^)	(6.4 ± 0.2 ^c^)	(9.2 ± 1.1 ^c^)	(6.7 ± 0.1 ^c^)
**3,4-COQ**	19.1 ± 0.4	22.3 ± 6.2	2.6 ± 0.1	2.9 ± 0.5	3.2 ± 0.0
	(37.0 ± 0.5 ^c^)	(43.1 ± 12.0 ^b^)	(5.1 ± 0.2 ^b^)	(5.7 ± 0.9 ^b^)	(6.2 ± 0.1 ^b^)
**3,5-COQ**	55.9 ± 2.6	60.2 ± 2590	3.4 ± 0.0	3.2 ± 0.5	3.6 ± 0.1
	(108.0 ± 5.1 ^f^)	(116.5 ± 5.0 ^c^)	(6.7 ± 0.1 ^c^)	(6.1 ± 0.9 ^b^)	(7.0 ± 0.3 ^c^)
**4,5-COQ**	4.3 ± 0.3	23.3 ± 0.3	2.6 ± 0.1	1.7 ± 0.9	2.8 ± 0.0
	(8.3 ± 0.3 ^b^)	(45.2 ± 0.6 ^b^)	(3.4 ± 2.9 ^a^)	(3.4 ± 1.8 ^a^)	(5.4 ± 0.1 ^a^)
**Trolox**	33.4 ± 0.5	26.8 ± 1.5	16.4 ± 2.9	6.2 ± 0.0	6.9 ± 0.1
	(0.1 ± 0.0 ^a^)	(0.1 ± 0.0 ^a^)	(31.7 ± 5.5 ^d^)	(12.0 ± 0.1 ^d^)	(13.4 ± 0.2 ^d^)

The IC_50_ value (in μg/mL unit) was defined as the final concentration of 50% radical inhibition or relative reducing power, calculated by linear regression analysis, and expressed as the mean ± SD (*n* = 3). The linear regression was analyzed by Origin 6.0 professional software. The IC_50_ value was also expressed in μM/mM unit. The IC_50_ value in the μM/mM unit, with different superscripts (a, b, c, d, e, or f) in the same diagram, are significantly different (*p* < 0.05). Trolox is the positive control. **1**,**3-COQ**: 1,3-di-*O*-caffeoylquinic acid; **1**,**5-COQ**: 1,5-di-*O*-caffeoylquinic acid; **3**,**4-COQ**: 3,4-di-*O*-caffeoylquinic acid; **3**,**5-COQ**: 3,5-di-*O*-caffeoylquinic acid; **4**,**5-COQ**: 4,5-di-*O*-caffeoylquinic acid. The dose-response curves are listed in Figure S1.

**Table 2:** The viability percentages of five **di-COQs** towards •OH-damaged bmMSCs in the MTT assay.

Compounds	Control	Model	10 μg/mL	30 μg/mL	50 μg/mL	100 μg/mL
**1,3-COQ**	100%	12.67%	17.03% *	20.23% *	23.97% *	47.15% *
**1,5-COQ**	100%	12.67%	19.09% *	21.65% *	24.90% *	44.93% *
**3,4-COQ**	100%	12.67%	13.02%	13.97%	15.53% *	21.15% *
**3,5-COQ**	100%	12.67%	13.06%	16.21% *	19.55% *	23.38% *
**4,5-COQ**	100%	12.67%	14.14%	21.78% *	22.72% *	42.04% *

Experiments were performed with 3 different batches of cells and each batch was tested in triplicate. The Fenton reagent (FeCl_2_ plus H_2_O_2_) was used to generate •OH radicals. These data represent the mean ± SD (*n* = 3). * *p* < 0.05 vs. model. MTT, 3-(4,5-dimethylthiazol-2-yl)-2,5-diphenyltetrazolium bromide. **1**,**3-COQ**: 1,3-di-*O*-caffeoylquinic acid; **1**,**5-COQ**: 1,5-di-*O*-caffeoylquinic acid; **3**,**4-COQ**: 3,4-di-*O*-caffeoylquinic acid; **3**,**5-COQ**: 3,5-di-*O*-caffeoylquinic acid; **4**,**5-COQ**: 4,5-di-*O*-caffeoylquinic acid.

## Supplementary Materials

The following are available online. Video S1–5: Animations of **1**,**3-COQ**, **1**,**5-COQ**, **3**,**4-COQ**, **3**,**5-COQ**, and **4**,**5-COQ**. Figure S1: Dose response curves of five **di-COQs** in various assays. Figure S2: Original Data of UPLC-ESI-MS/MS analysis.

## Abbreviations

The following abbreviations are used in this manuscript:

**1,3-COQ**	1,3-di-*O*-caffeoylquinic acid
**1,5-COQ**	1,5-di-*O*-caffeoylquinic acid
**3,4-COQ**	3,4-di-*O*-caffeoylquinic acid
**3,5-COQ**	3,5-di-*O*-caffeoylquinic acid
**4,5-COQ**	4,5-di-*O*-caffeoylquinic acid
ABTS	[2,2′-azino-bis(3-ethylbenzo-thiazoline-6-sulfonic acid diammonium salt)]
bmMSCs	bone marrow-derived mesenchymal stem cells
DMEM	Dulbecco’s modified Eagle’s medium
DPPH•	(1,1-diphenyl-2-picryl-hydrazl)
ET	electron transfer
FBS	fetal bovine serum
FRAP	ferric reducing antioxidant power
HAT	hydrogen atom transfer
PTIO•	2-phenyl-4,4,5,5-tetramethylimidazoline-1-oxyl 3-oxide radical
ROS	reactive oxygen species
RNS	reactive nitrogen species
RAF	radical adduct formation
SD	standard deviation
TPTZ	2,4,6-tris(2-pyridyl-s-triazine)
Trolox	[(±)-6-hydroxyl-2,5,7,8-tetramethlychromane-2-carboxylic acid]
