# Supplementary material for: Antioxidant and Cytoprotective Effects of the Di-O-Caffeoylquinic Acid Family: The Mechanism, Structure–Activity Relationship, and Conformational Effect
Source: Molecules. 2018 Jan 20;23(1):222. doi: 10.3390/molecules23010222 (PMC6017143; doi:10.3390/molecules23010222)
Supplement: Supplementary file 1 [file molecules-23-00222-s001.zip › molecules-255685-supplementary/Suppl. 1-7/Fig. S1-2 HPLC-MS.pdf]

Supplements

Antioxidant and Cytoprotective Effects of the Di-*O*-Caffeoylquinic Acid Family: The Mechanism, Structure–Activity Relationship, and Conformational Effect

Xican Li [1,2,*,†], Ke Li [3,4,†], Hong Xie [1,2], Yulu Xie [1,2], Yueying Li [1], Xiaojun Zhao [1,2], Xiaohua Jiang [5] and Dongfeng Chen [3,4,*]

1 School of Chinese Herbal Medicine, Guangzhou University of Chinese Medicine, Waihuan East Road No. 232, Guangzhou Higher Education Mega Center, Guangzhou 510006, China; xiehongxh1@163.com (H.X.); xieyulu1900@163.com (Y.X.); lln@gzucm.edu.cn (Y.L.); zxj@gzucm.edu.cn (X.Z.)

2 Innovative Research & Development Laboratory of TCM, Guangzhou University of Chinese Medicine, Waihuan East Road No. 232, Guangzhou Higher Education Mega Center, Guangzhou 510006, China

3 School of Basic Medical Science, Guangzhou University of Chinese Medicine, Waihuan East Road No. 232, Guangzhou Higher Education Mega Center, Guangzhou 510006, China; 1090992678@qq.com (K.L.)

4 The Research Center of Basic Integrative Medicine, Guangzhou University of Chinese Medicine, Waihuan East Road No. 232, Guangzhou Higher Education Mega Center, Guangzhou 510006, China

5 School of Biomedical Sciences, Faculty of Medicine, The Chinese University of Hong Kong, Sha Tin, Hong Kong, China; xjiang@cuhk.edu.hk (X.J.)

* Correspondence: lixican@126. com (X.L.); chen888@gzucm.edu.cn (D.C.); Tel.: +86-203-935-8076
† These authors contributed equally to this work.

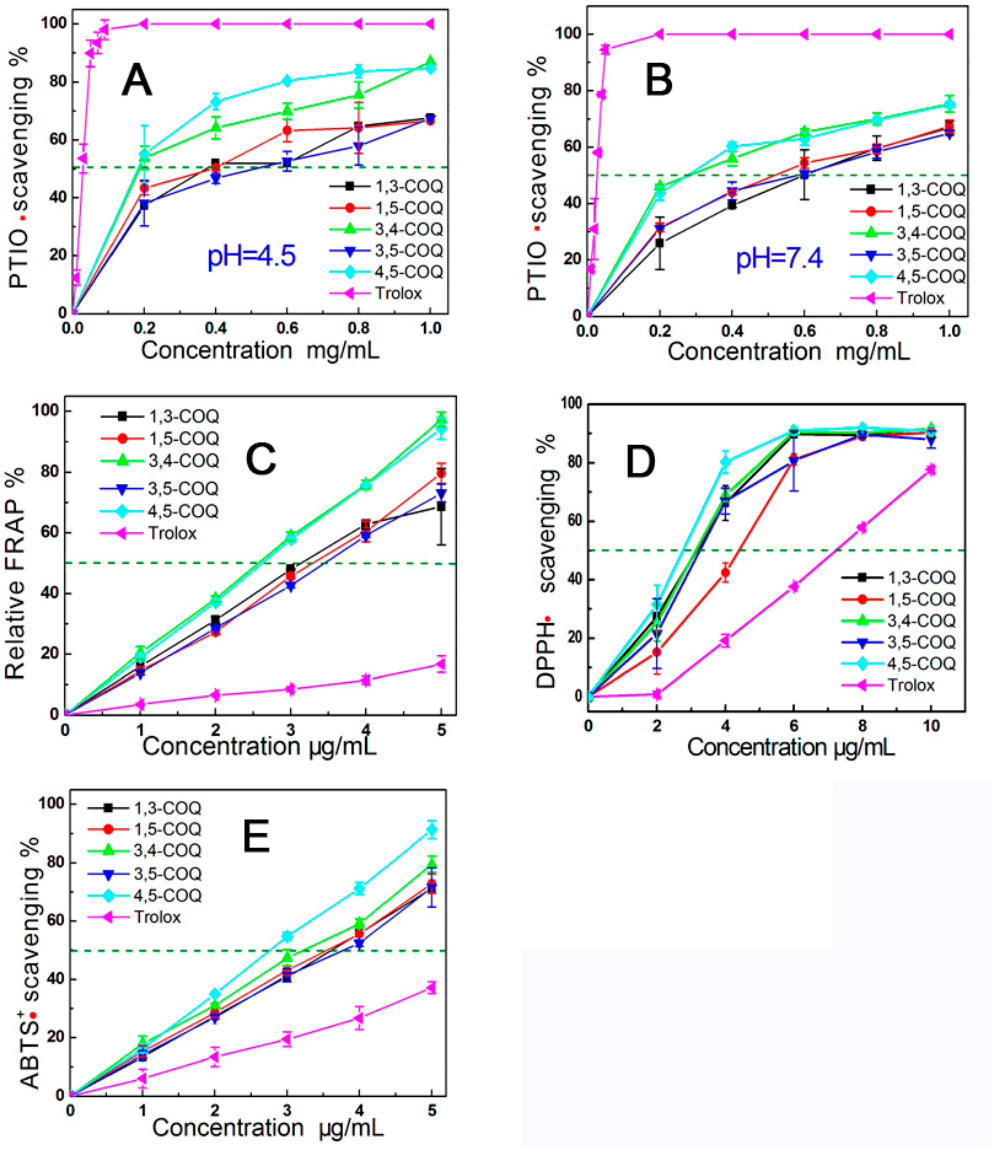

Fig. S1. Dose response curves of di-*O*-caffeoylquinic acids (**di-COQs**) in various antioxidant assays: (A) PTIO•-radical-scavenging assay (pH 4.5); (B) PTIO•-radical-scavenging assay (pH 7.4). (C) FRAP assay. (D) DPPH•-scavenging assay. (E) $ABTS^{+}$•- scavenging assay.

Each value is expressed as the mean±SD, *n*=3.

Spectrum of PTIO•

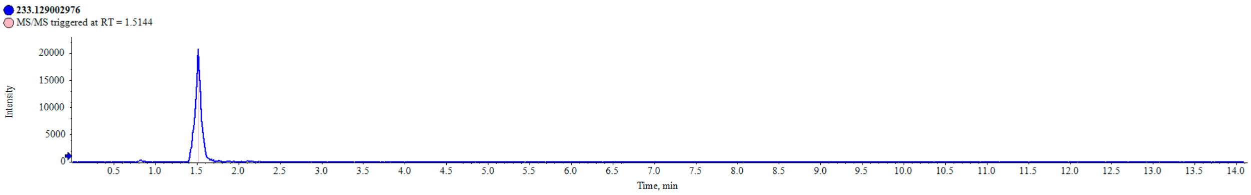

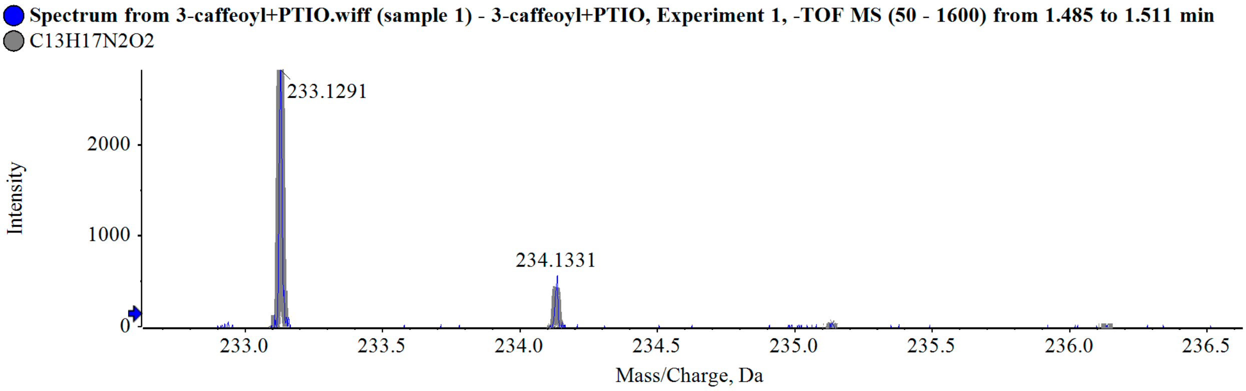

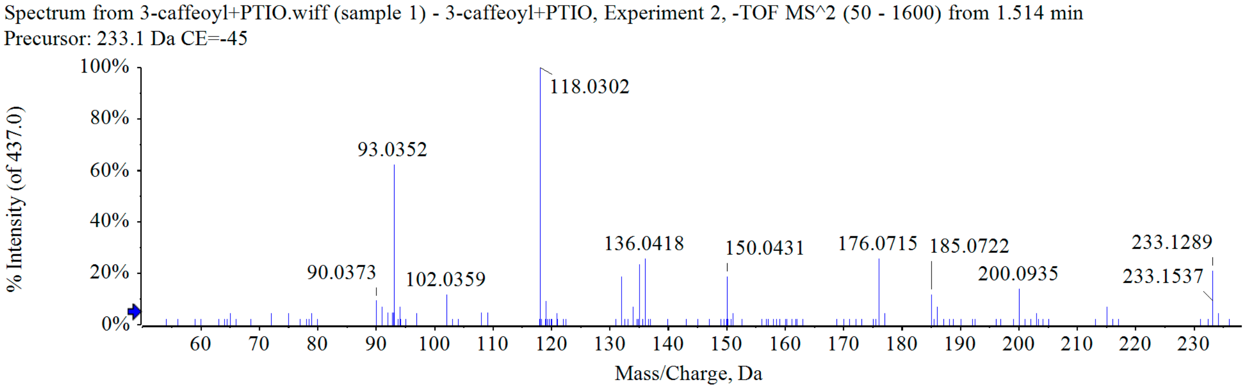

Spectrum of Dimer product in the mixture of chlorogenic acid with PTIO •

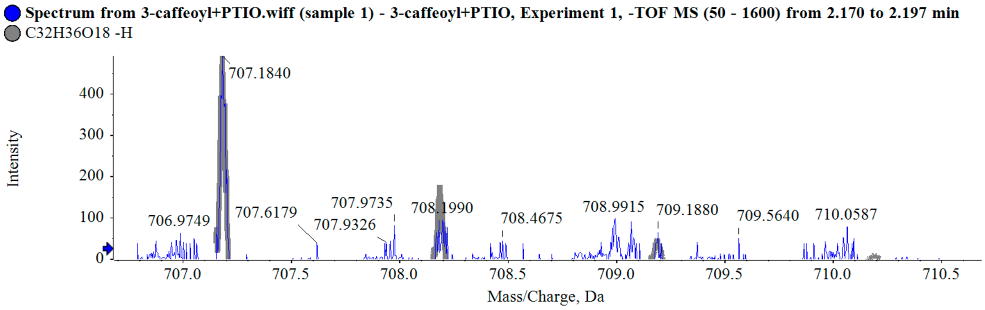

Fig. S2 **Original Data of UPLC-ESI-MS/MS analysis.**
